# Quantitative Analysis of Choriocapillaris Using Swept-Source Optical Coherence Tomography Angiography in Eyes with Angioid Streaks

**DOI:** 10.3390/jcm11082134

**Published:** 2022-04-11

**Authors:** Hoang Mai Le, Eric H. Souied, Safa Halouani, Enrico Borrelli, Thibaut Chapron, Giuseppe Querques, Alexandra Miere

**Affiliations:** 1 Department of Ophthalmology, Centre Hospitalier Intercommunal de Créteil, 94000 Créteil, France; hoangmai.le@chicreteil.fr (H.M.L.); eric.souied@chicreteil.fr (E.H.S.); safa.halouani@chicreteil.fr (S.H.); 2 Department of Ophthalmology, IRCCS Ospedale San Raffaele, 20132 Milan, Italy; borrelli.enrico@yahoo.com (E.B.); giuseppe.querques@hotmail.it (G.Q.); 3 Fondation Ophtalmologique Adolphe de Rothschild, 75019 Paris, France; tibochapron@hotmail.com

**Keywords:** angioid streaks, optical coherence tomography-angiography, choriocapillaris, retina

## Abstract

Purpose: to quantitatively analyze choriocapillaris perfusion using swept-source optical coherence tomography angiography (SS-OCTA) in eyes presenting with angioid streaks in comparison with control eyes. Methods: Macular 6 × 6 mm SS-OCTA scans were retrospectively analyzed in eyes with angioid streaks and in control eyes. En face choriocapillaris flow images were compensated with en face choriocapillaris structure images, followed by the Phansalkar local thresholding method (with a window radius of four and eight pixels). Quantitative analysis was performed in the four peripheral 1 × 1 mm corners of the 6 × 6 mm SS-OCTA image to include equidistant and comparable regions. The percentage of flow deficits (FD%), the number and size of the flow deficits (FDs) and the total area of FDs were then calculated. Results: 54 eyes of 31 patients were included in the study: 27 eyes diagnosed with angioid streaks and 27 controls. Analysis of the four 1 × 1 mm peripheral corners of the 6 × 6 mm SS-OCTA image showed that eyes with angioid streaks had a higher FD% compared to the control group (47.62 ± 8.06 versus 38.90 ± 6.38 using a radius of four pixels (*p* < 0.001); 48.37 ± 7.65 versus 39.66 ± 6.51 using a radius of eight pixels (*p* < 0.001). The average size of FDs as well as the total area size of the FDs were significantly higher in eyes with angioid streaks compared to control eyes (*p* < 0.001). Eyes with angioid streaks present reduced choriocapillaris flow compared to control eyes. Decreased choriocapillaris perfusion may contribute, among other factors, to the development of neovascularization and atrophy in patients with angioid streaks.

## 1. Introduction

Angioid streaks are described as irregular bilateral dark-red-to-gray jagged break lines, originating from the optic disc and radiating towards the periphery [1]. Histologically, they represent defects in the calcified Bruch’s membrane and can be associated with atrophy of the overlying retinal pigment epithelium (RPE) and choriocapillaris [2]. Several systemic diseases have been associated with angioid streaks, such as pseudoxanthoma elasticum, Ehlers–Danlos syndrome, hemoglobinopathies and Paget disease. Angioid streaks may be asymptomatic, but the development of macular neovascularization (MNV), occurring in approximately 42% to 86% of patients during follow-up, and macular atrophy, contributes to severe visual impairment in these patients [3,4]. Moreover, the onset of secondary MNV complicating angioid streaks occurs in the working age population, within the 5th decade according to recent literature [4,5]. While the prevalence of MNV and atrophy is directly proportional to the patients’ age, it has recently been shown that in patients with pseudoxanthoma elasticum, eyes with longer angioid streaks (i.e., more extensive calcification of Bruch’s membrane) have a higher risk of developing neovascularization or atrophy, and thereby will have a worse visual prognosis [6]. Of course, visual prognosis in these patients is dependent on the location of the angioid streaks (foveal or extrafoveal) and on the secondary neovascularization emanating from the growth of (fibro-) vascular tissue through the localized Bruch’s membrane defect. As the integrity of Bruch’s membrane is interrupted, this may lead to a change in growth factors interacting with a potential communication between the retina and the inner choroid (i.e., choriocapillaris), and consequent development of neovascularization [7].

Multimodal imaging can help identifying foveal or extrafoveal neovascularization associated with angioid streaks. Optical coherence tomography angiography (OCTA) has been shown to be useful not only in the detection of neovascular complications in patients with angioid streaks, but also in evaluating MNV activity [4,5]. Furthermore, OCTA may also help the clinicians gain insight into the very determinants of neovascularization and macular atrophy. Indeed, OCTA allows a high-resolution in vivo visualization of the choriocapillaris’ planar topology, with densely packed choriocapillaris and intertwined nonperfused areas, termed signal voids/flow deficits. The choriocapillaris perfusion has been showed to be decreased in several conditions, such as age-related macular degeneration, diabetic retinopathy, central serous chorioretinopathy, Vogt–Koyanagi–Harada disease, retinitis pigmentosa and idiopathic retinal membrane.

A growing body of literature shows that a decrease in choriocapillaris perfusion could lead to the development of neovascularization and atrophy in age-related macular degeneration [8,9].

As the development of neovascularization and atrophy play an important role in the visual prognosis of eyes with angioid streaks, the study of choriocapillaris perfusion (and subsequent nutrition of the outer retina via the choriocapillaris) in these patients could be of interest. Based on our information, previous studies have not focused on the choricapillaris quantitative analysis in eyes with angioid streaks using swept-source (SS) OCTA. Hence, the objective of the study is to quantitatively analyze the choriocapillaris alterations using SS-OCTA in eyes presenting with angioid streaks and to compare them with control eyes.

## 2. Materials and Methods

### 2.1. Description of the Study Patients

This study was a retrospective observational study. Data were collected from patients diagnosed with angioid streaks at Creteil Hospital (Department of Ophthalmology) between 2014 and 2021 who had undergone a 6 × 6 mm SS-OCTA during their follow-up in the department. Diagnosis of type of angioid streaks was made by fundus examination. This study was conducted in accordance with the Declaration of Helsinki. Because of the retrospective nature of the study, patient consent was waived.

Subjects had to have undergone both spectral-domain optical coherence tomography (SD-OCT) (Spectralis; Heidelberg Engineering, Heidelberg, Germany) and SS-OCTA (Plex Elite, Carl Zeiss Meditec, Dublin, CA, USA) in the same visit.

Exclusion criteria were: (1) an OCTA signal strength index below seven; (2) incorrect segmentation or significant motion artifact; (3) other associated retinal diseases in the study eye.

Demographic data (including age and sex) were collected for all patients. Best-corrected visual acuity (BCVA) assessment, slit-lamp and fundus examination were realized for all patients. Multimodal imaging was performed for all patients including infrared imaging (IR), fundus autofluorescence (FAF), SD-OCT and SS-OCTA. For patients with macular neovascularization (MNV), the number of past intravitreal injections of anti-vascular endothelial growth factor (anti-VEGF) was noted. Association with systemic diseases was also noted.

### 2.2. Imaging and Image Processing

Patients underwent a 6 × 6-mm SS-OCTA scan that was centered on the fovea. Both the choriocapillaris en face structure and choriocapillaris *en face* flow images were extracted from the SS-OCTA machine. In order to segment the choriocapillaris slab, we used manual segmentation as previously described by Chu et al. The slab was 15 µm thick and started 16 µm below the RPE/BM [10].

When present, segmentation errors were corrected manually. Fiji Software (National Institute of Mental Health, Bethesda, MD, USA) was used to import the images. The signal attenuation induced by deposits onto the choriocapillaris was compensated using a formerly described method of compensation [11,12].

A compensated en face choriocapillaris image was therefore created. The compensated en face choriocapillaris flow image was then binarized using the Phansalkar local thresholding method in order to obtain a quantitative analysis of the flow deficits as previously reported in the literature. A window of both four pixels and eight pixels of radius were used [12].

**Figure 1:**
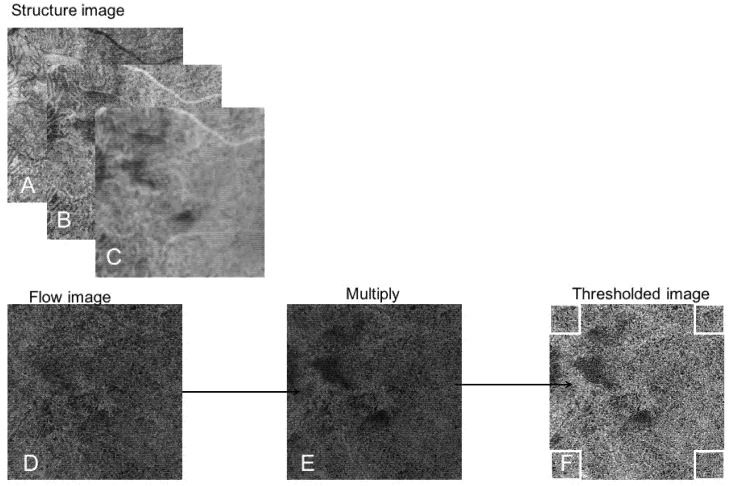
Image processing methodology used for quantification of the choriocapillaris flow. En face choriocapillaris structure image (**A**) was transformed using the Fiji “Invert” function (**B**). Gaussian blur filter was then used for smoothing (**C**). The en face choriocapillaris flow image (**D**) was multiplied with the resulting en face choriocapillaris structure image (**C**) with the “Image Calculator” function. A compensated en face choriocapillaris image was created (**E**). The compensated en face choriocapillaris flow image was then binarized, to quantitatively measure the flow deficits as reported in the current literature (**F**). Analysis was performed within the four corners of the OCTA 6 × 6 mm images measuring each 1 × 1 mm. This analysis was performed in order to include comparable and equidistant regions outside of the area of choroidal neovascularization, when present.

The window radius of four pixels equaled 26.37 microns and the window radius of eight pixels equaled 49.80 microns. Analysis was realized within the four corners of the 6 × 6 mm SS-OCTA images: Four squares measuring 1 × 1 mm were selected at the four corners of the images as described in recent literature [13] to include comparable and equidistant areas in all eyes outside of the area of choroidal neovascularization, when present. Squares were located (1) in the superonasal corner (2) in the superotemporal corner (3) in the inferonasal corner and (4) in the inferotemporal corner. The percentage of flow deficits (FD%), number and size of flow deficits (FDs) and total FDs area were measured with the “Analyze Particles” function (size: 0-infinity, circularity 0–1) [12]. The average of FD%, number of FDs and total FDs area of the four squares was determined for each eye. Figure 1 illustrates the methodology of the analysis.

### 2.3. Statistical Methodology

All qualitative variables were reported in percentages. All quantitative variables were reported by their mean with their standard deviation. The Wilcoxon signed-rank test was used to compare the quantitative variables.

## 3. Results

### 3.1. Patient Demographics and Clinical Characteristics

54 eyes of 31 patients were analyzed in the study: 27 eyes diagnosed with angioid streaks (14 patients) and 27 controls (17 patients).

Mean age was 64 ± 14.92 years in the angioid streaks group and 63.92 ± 8.39 years in the control group. There was no statistical difference regarding age between the two groups (*p* > 0.05). Best corrected visual acuity (BCVA) was 0.42 ± 0.49 (logarithm of the minimum angle of resolution (20/50 Snellen equivalent) in the angioid streaks group (median 0.2, IQR 0.85) and 0 ± 0 (20/20 Snellen equivalent) in the control group (*p* > 0.05).

In the angioid streaks group, three of the patients had an associated confirmed systemic disease (two pseudoxanthoma elasticum and one sickle cell disease). MNV was associated in 21 of the eyes with angioid streaks. In eyes with angioid streaks associated with MNV, mean number of past intravitreal anti-VEGF injections was 13.13 ± 9.31.

### 3.2. Choriocapillaris Quantitative Study of the Peripheral 1 × 1 mm Squares Using a Window Radius of Four Pixels (Phansalkar Local Thresholding Method)

**Figure 2:**
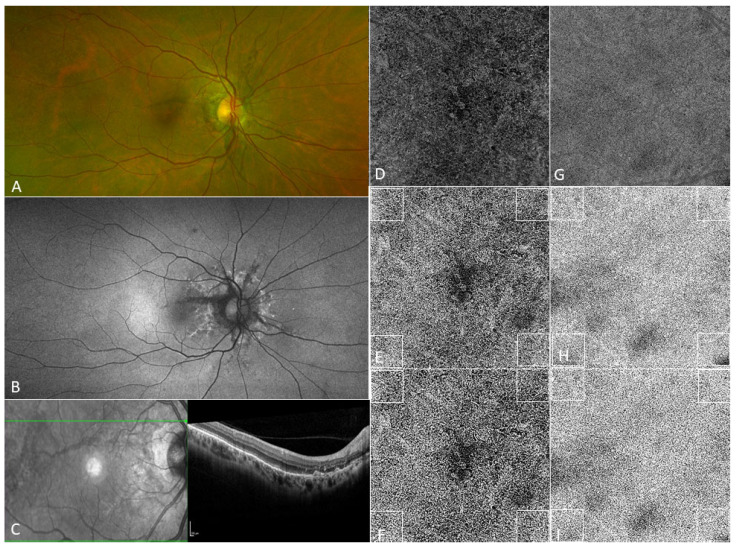
Quantitative analysis of the choriocapillaris and multimodal imaging of a 55 year-old patient presenting with angioid streaks. (**A**): Ultra-wide field color fundus photography revealing the presence of grey peripapillary jagged break lines. (**B**): Ultra-wide field autofluorescence fundus photography in the same patient showing hypo- and hyperautofluorescent break lines radiating from the optic disc. (**C**): Spectral domain optical coherence tomography in the same patient showing peripapillary atrophy. (**D**): Choriocapillaris en face flow image of the patient in panels A–C, as well as the resulting image after image processing using the Phansalkar local thresholding method with a window radius of four pixels (**E**) and eight pixels (**F**). Of note, the right panel shows the images of a 57-year-old healthy control choriocapillaris en face (**G**) flow image as well as the resulting image after image processing using the Phansalkar local thresholding method with a window radius of four pixels (**H**) and eight pixels (**I**).

The analysis was performed within the four 1 × 1 mm squares situated at the four corners of the 6 × 6 mm SS-OCTA image, as shown in Figure 2, in order to include comparable and equidistant areas without choroidal neovascularization in the studied eyes as reported in current literature [13]. Analysis was first performed using a window radius of four pixels.

Mean average choriocapillaris FD% of the four peripheral 1 × 1 mm squares was increased in the angioid streaks group (47.62 ± 8.06) compared to the control group (38.90 ± 6.38) with statistical significance (*p* < 0.001). Mean average total area of FDs of the four peripheral 1 × 1 mm squares was also significantly increased in the angioid streaks group 0.47 ± 0.08 mm^2^ compared to the control group 0.39 ± 0.06 mm^2^ (*p* < 0.001). Mean average size of FDs of the four peripheral 1 × 1 mm squares was 5312.99 ± 4489.96 μm^2^ in angioid streaks patients versus 2784.18 ± 6848.48 μm^2^ in control patients (*p* < 0.001). The average number of FDs of the four peripheral 1 × 1 mm squares was significantly reduced (202.45 ± 125.28) in the angioid streaks group compared to the control group (364.12 ± 125.28) (*p* < 0.001).

Table 1 summarizes these results.

### 3.3. Choriocapillaris Quantitative Study of the Peripheral 1 × 1 mm Squares Using a Window Radius of Eight Pixels (Phansalkar Local Thresholding Method)

A second analysis was performed with a window radius of eight pixels, as shown in Figure 2. Mean choriocapillaris FD% of the four peripheral 1 × 1 mm squares was increased in the angioid streaks group (48.37 ± 7.65) in comparison to the control group (39.66 ± 6.51) with statistical significance (*p* < 0.001). The average total area of FDs of the four peripheral 1 × 1 mm squares was significantly increased in the angioid streaks group 0.48 ± 0.08 mm^2^ compared to the control group 0.39 ± 0.06 mm^2^ (*p* < 0.001). Mean average size of FDs of the four peripheral 1 × 1 mm squares was 5496.93 ± 4491.52 μm^2^ in angioid streaks patients versus 2758.13 um^2^ ± 5953.73 in control patients (*p* < 0.001). Mean number of FDs of the four peripheral 1 × 1 mm squares was 160.11 ± 130.83 in the angioid streaks group and 317.88 ± 118.47 in the control group (*p* = 0.38). Table 1 summarizes the results.

### 3.4. Choriocapillaris Quantitative Study of the Peripheral 1 × 1 mm Squares Depending on Their Location

Choriocapillaris FD% was compared for each 1 × 1 mm square depending on their location. Mean choriocapillaris FD% was of 46.19 ± 9.52 using a radius of four pixels and 47.06 ± 8.96 using a radius of eight pixels in the inferonasal square in the angioid streaks group and significantly increased (*p* < 0.05) compared to the mean choriocapillaris FD% in the control group in the same square (40.43 ± 11.29 using a radius of four pixels and 41.21 ± 11.121 using a radius of eight pixels). Similarly, mean choriocapillaris FD% was significantly increased in the angioid streaks group compared to the control group in the inferotemporal square, the superonasal square and the superotemporal square (*p* < 0.001). Table 2 summarizes the results.

### 3.5. Choriocapillaris FD% in Angioid Streaks with versus without MNV

A secondary analysis was performed in order to compare FD% between eyes with angioid streaks associated with MNV and eyes with angioid streaks without MNV. There was no statistically significant difference in FD%, using a radius of both four and eight pixels, between the group of patients with angioid streaks with MNV (FD%= 48.02 ± 6.85; FD% = 48.76 ± 6.40 using a radius of four and eight pixels, respectively) and the group without MNV (FD = 46.20 ± 12.11; FD% = 7.70 ± 11.71 using a radius of four and eight pixels, respectively), using the Mann–Whitney–Wilcoxon test; *p* = 0.84 and *p* =0.80 with a radius of four and eight pixels, respectively.

## 4. Discussion

In this retrospective observational study, choriocapillaris flow deficits were quantitatively analyzed in eyes presenting with angioid streaks using SS-OCTA in comparison with control eyes. We noted that eyes with angioid streaks presented a decreased choriocapillaris perfusion, with a significantly increased FD%, total area of FDs and average size of the FDs compared to control eyes when analyzing the peripheral 1 × 1 mm of the SS-OCTA images. However, there was no significant difference in terms of FD% between eyes with angioid streaks with and without secondary MNV. To our knowledge, this is the first study quantitatively analyzing choriocapillaris flow deficits in patients with angioid streaks.

Choriocapillaris perfusion has been shown to be reduced in several retinal diseases. Several authors have hypothesized that a reduced choriocapillaris perfusion and dysfunction of the RPE-Bruch’s membrane-choriocapillaris complex can play a role in the development of neovascularization and atrophy, especially in AMD patients. [14,15,16]

Angioid streaks, which are visible as grey jagged lines radiating from the optic disc, constitute irregular crack-like dehiscence of the calcified, brittle Bruch’s membrane. The Bruch’s membrane, which is attached to the RPE, is an elastin- and collagen-rich membrane involved in the transportation of nutrients and metabolites between the RPE and the choriocapillaris [17].

In eyes with angioid streaks, mineralization of the Bruch’s membrane occurs, resulting in a deposition of calcium in Bruch’s membrane which may impair oxygen diffusion from the choroid to the outer retina [17] Furthermore, a loss of integrity of the Bruch’s membrane would not only lead to a change of angiogenic growth factors, but also allow the growth of (neo-) vessels emanating from the choroid through the mechanical defect [7]. In addition to the mechanical break, reduced choriocapillaris perfusion in these eyes, as shown by the results of the present study, may contribute to outer retina ischemia, resulting in an even more increased secretion of VEGF and the development of neovascularization [6,7,18]. As the macular area has a higher oxygen demand, the 6 × 6 mm OCTA image was analyzed overall, but also the 1 × 1 mm peripheral squares of the image, in order to avoid bias due to the presence of secondary MNV in some of the included eyes. Interestingly, in addition to a higher FD% in eyes with angioid streaks compared to healthy controls, there was no significant difference in FD% between eyes with angioid streaks with and without secondary MNV. Hence, it would be plausible to suggest that choriocapillaris impairment is only one of the factors triggering the growth of neovascularization and/or atrophy. Moreover, eyes with angioid streaks can develop atrophy of the RPE, contributing to visual loss depending on its location. Several studies showed that choriocapillaris perfusion is reduced in eyes with AMD, and even more strikingly in patients with geographic atrophy [18,19]. Decreased choriocapillaris perfusion and choroidal degeneration could impair the clearance of lipid debris from the RPE/Bruch’s membrane and hypoxia, impacting the neural retina and the RPE. 

Many factors may influence the analysis of the choriocapillaris perfusion using OCTA. A swept-source OCTA, compared to a spectral domain OCTA, has the advantage of using a longer wavelength of 1050, allowing a deeper light penetration into the choroid as well as a better determination of macular neovascularization when present. In our study, we used a method of compensation to reduce signal attenuation generated by the RPE-Bruch’s membrane complex on the choriocapillaris, as described previously [12,16] Slab selection should also be performed carefully when analyzing the choriocapillaris perfusion. In our study, we used a slab of a thickness of 15 µm, starting 16 µm under the RPE or Bruch’s membrane as described by Chu et al. [10]. Indeed, automated segmentation is sometimes not accurate in diseased eyes due to incorrect RPE identification. The thresholding method can also influence the results in quantitative choriocapillaris analysis. It has been demonstrated that using the Phansalkar local thresholding method, a local window radius measuring 1–2 times the ICD, corresponding to four to eight pixels, is more accurate [10]. We therefore used a local window radius of four and eight pixels.

This study has some limitations; firstly, its cross-sectional and retrospective nature. A longitudinal and prospective study could help to better clarify the relation between a reduced choriocapillaris perfusion and development of neovascularization and atrophy. Secondly, in the angioid streaks group, some eyes presented with MNV while others did not. However, as we decided to analyze the choriocapillaris flow in the 1 × 1 mm peripheral squares, we excluded the area of neovascularization. Finally, eyes presenting with angioid streaks associated with neovascularization were treated with anti-VEGF intravitreal injections, which could impact the results. However, no previous OCTA studies have described significant changes in choroidal thickness, nor in the choriocapillaris flow, before and after anti-VEGF intravitreal injections [20].

## 5. Conclusions

In conclusion, we found that eyes presenting with angioid streaks have a reduced choriocapillaris perfusion compared to control eyes, with an increased FD% and a higher total area and average size of FDs. This decrease in choriocapillaris flow may contribute, among other factors, to the development of neovascularization and atrophy in angioid streaks eyes. Further studies are needed to better evaluate the relationship between choriocapillaris perfusion and the presence of angioid streaks.

## Figures and Tables

**Table 1:** Choriocapillaris quantitative study of the peripheral 1 × 1 mm squares applying the Phansalkar local thresholding method: comparisons between angioid streaks eyes and control eyes.

	Angioid Streaks Eyes (*n* = 27)	Control Eyes (*n* = 27)	*p*
R4 Mean percentage of flow deficits (FD%)	47.62 ± 8.06	38.90 ± 6.38	*p* < 0.001 *
R4 Mean total area of flow deficits (mm^2^)	0.47 ± 0.08	0.39 ± 0.06	*p* < 0.001 *
R4 Mean size of flow deficits (μ > m^2^)	5312.99 ± 4489.96	2784.18 ± 6848.48	*p* < 0.001 *
R4 Mean number of flow deficits	202.45 ± 125.28	364.12 ± 125.28	*p* < 0.001 *
R8 Mean percentage of flow deficits (FD%)	48.37 ± 7.65	39.66 ± 6.51	*p* < 0.001
R8 Mean total area of flow deficits (>mm^2^)	0.48 ± 0.08	0.39 ± 0.06	*p* < 0.001
R8 Mean size of flow deficits (μ > m^2^)	5496.93 ± 4491.52	2758.13 ± 5953.73	*p* < 0.001
R8 Mean number of flow deficits	160.11 ± 130.83	317.88 ± 118.47	*p* = 0.38

R4: window radius of four pixels. R8: window radius of eight pixels. *: Wilcoxon signed-rank test was used for statistical analysis. Quantitative values are noted in mean ± standard deviation.

**Table 2:** Choriocapillaris Quantitative Study of the Peripheral 1 × 1 mm squares applying the Phansalkar local thresholding method: comparisons between angioid streaks eyes and control eyes.

	Angioid Streaks Eyes (*n* = 27)	Control Eyes (*n* = 27)	*p*
R4 FD% in the superonasal quadrant	48.38 ± 9.52	38.87 ± 6.48	*p* < 0.001 *
R4 FD% in the inferonasal quadrant	46.19 ± 9.52	40.43 ± 11.29	*p* < 0.03 *
R4 FD% in the superotemporal quadrant	48.38 ± 9.52	38.87 ± 6.48	*p* < 0.001 *
R4 FD% in the inferotemporal quadrant	47.87 ± 12.58	38.61 ± 6.86	*p* < 0.01 *
R8 FD% in the superonasal quadrant	49.04 ± 9.01	39.56 ± 6.35	*p* < 0.001
R8 FD% in the inferonasal quadrant	47.96 ± 8.96	41.21 ± 11.12	*p* < 0.001
R8 FD% in the superotemporal quadrant	49.04 ± 9.01	39.57 ± 6.34	*p* < 0.001
R8 FD% in the inferotemporal quadrant	48.62 ± 11.91	39.35 ± 6.61	*p* < 0.01 *

FD%: Percentage of flow deficits. R4: window radius 4 of pixels. R8: window radius 8 of pixels. *: Wilcoxon signed-rank test was used for statistical analysis. Quantitative values are noted in mean ± standard deviation.

## Data Availability

Data are available upon reasonable request from the corresponding authors.
